# Mitochondrial dysfunction in Alzheimer’s disease: targeting the powerhouse with nanomedicine

**DOI:** 10.3389/fphar.2026.1755126

**Published:** 2026-05-29

**Authors:** Chen Kang, Xue Zhou, Bingchen Li, Jiamin Li

**Affiliations:** Division of Neurology, Affiliated Hospital of Shandong University of Traditional Chinese Medicine, Jinan, China

**Keywords:** Alzheimer’s disease, blood-brain barrier, mitochondrial dysfunction, nanomedicine, targeted therapy

## Abstract

Alzheimer’s disease (AD) is a progressive neurodegenerative disorder characterized by relentless cognitive decline. Despite decades of research dominated by the amyloid and tau hypotheses, clinical interventions targeting these classical hallmarks have yielded limited success in halting disease progression, underscoring a critical conceptual and therapeutic gap: the early, persistent, and under-addressed role of mitochondrial dysfunction as a primary driver of AD pathology. Beyond serving as passive victims of amyloid-beta toxicity, impaired mitochondria actively initiate and perpetuate a self-amplifying cycle through dysregulated bioenergetics, aberrant dynamics, compromised quality control, calcium dyshomeostasis, and exacerbated neuroinflammation-all of which collectively propel synaptic loss and neuronal death well before overt plaque deposition. This review provides a synthesized reappraisal of this mitochondrial-centric paradigm, systematically delineating the interconnected nature of mitochondrial failure as both an initiator and an amplifier of AD and arguing that it represents a central nexus linking sporadic and genetic forms of the disease. We further consolidate the emerging landscape of nanomedicine as a revolutionary strategy to bridge the current therapeutic gap, highlighting how diverse nanotherapeutic platforms-ranging from organic and inorganic nanocarriers and biomimetic vesicles to nanozymes and gene-modulating systems-are uniquely equipped to overcome the blood-brain barrier and achieve subcellular precision targeting of the mitochondrial compartment. By analyzing advances in nanotechnology designed explicitly to restore mitochondrial homeostasis through the normalization of redox balance, enhancement of mitophagy, and preservation of adenosine triphosphate synthesis, we demonstrate how this approach directly addresses the root of the neurodegenerative cascade that traditional drug development has failed to reach. Finally, we critically examine the translational hurdles and future trajectories of mitochondria-targeted nanotherapy, proposing a conceptual framework for multidisciplinary integration and arguing that mitochondrial nanomedicine represents not merely a symptomatic intervention but a requisite disease-modifying paradigm shift capable of intercepting the earliest triggers of AD.

## 1 Introduction

Alzheimer’s disease (AD) is the most common cause of dementia and is characterized clinically by progressive impairment of memory, executive function, and daily living abilities. Its neuropathological hallmarks are extracellular Aβ plaques and intracellular neurofibrillary tangles composed mainly of hyperphosphorylated tau (Blackstone et al., 2021; Lukens and Williams, 2022; Lim et al., 2024). However, amyloid and tau pathology alone do not fully account for the timing, regional vulnerability, metabolic decline, synaptic failure, or therapeutic resistance observed in AD (Doty, 2017; Samuels and Lukens, 2025; Small et al., 2011). This has prompted increasing attention to upstream and parallel mechanisms, among which mitochondrial dysfunction is particularly important.

The mitochondrial cascade hypothesis proposes that inherited and acquired variation in mitochondrial function may influence AD risk, age at onset, and disease trajectory (Yang L. et al., 2020; Kamienieva et al., 2021). In this framework, mitochondrial bioenergetic decline, oxidative stress, impaired calcium handling, disrupted mitochondrial dynamics, and defective mitochondrial quality control can precede or accompany Aβ and tau pathology. Aβ has been reported to accumulate in mitochondria and to interact with proteins such as amyloid-beta-binding alcohol dehydrogenase (ABAD), whereas pathological tau can impair axonal mitochondrial trafficking and promote synaptic energy failure. Mitochondria, the primary energy producers of cells, are essential for maintaining cellular function and survival (Ravindran and Gustafsson, 2025; Wang H. et al., 2024; Wang S. et al., 2023). Through oxidative phosphorylation (OXPHOS), they generate ATP, the energy currency required for cellular activities (Nolfi-Donegan et al., 2020; Christofides et al., 2021). Concurrently, the tricarboxylic acid (TCA) cycle not only sustains energy production but also provides essential substrates for biosynthesis and helps maintain intracellular redox balance (Corbet et al., 2016). Disruption of OXPHOS and dysregulation of the TCA cycle are implicated in a spectrum of brain diseases, underscoring the critical importance of mitochondrial health for overall brain function (Chan, 2020; Skawratananond et al., 2025; McGill Percy et al., 2025). Beyond their role in energy metabolism, mitochondria also serve as crucial signaling hubs, regulating multiple pathways essential for neuronal health and function (Pahal et al., 2025).

The therapeutic significance of this perspective is that restoring mitochondrial homeostasis may modify several AD-relevant processes simultaneously, including ATP production, reactive oxygen species (ROS) generation, calcium buffering, apoptosis, mitophagy, neuroinflammation, and synaptic maintenance (Goedert, 2015). Conventional small molecules and antioxidants have often shown limited clinical benefit, partly because they do not efficiently reach the brain, accumulate in vulnerable neuronal mitochondria, or sustain activity in the complex AD microenvironment (Liu B.-H. et al., 2024; Abramov and Bachurin, 2021). Therefore, the development of strategies that can both cross the blood-brain barrier (BBB) and achieve mitochondrial localization is a compelling but technically demanding direction.

In this review, we first summarize AD-relevant mitochondrial biology with emphasis on the mitochondrial features that are directly involved in disease mechanisms. We then critically discuss mitochondrial dysfunction in AD pathogenesis, including energy metabolism, oxidative stress, calcium dyshomeostasis, mitochondrial dynamics, mitophagy, mitochondria-associated endoplasmic reticulum membranes (MAMs), Aβ/tau interactions, and neuroinflammation. Next, we evaluate BBB traversal and mitochondrial targeting as linked delivery challenges. Finally, we synthesize recent nanomedicine strategies for mitochondrial targeting in AD and discuss their translational barriers. The aim is not merely to catalogue nanosystems, but to define which mitochondrial defects they target, how convincing the evidence is, and what must be resolved before clinical translation.

## 2 AD-relevant mitochondrial biology: structures and functions that become therapeutic targets

Mitochondria are double-membrane organelles whose disease relevance in AD is best understood through the functions of specific compartments rather than through a detailed textbook description of organelle anatomy (Leung et al., 2021; de Araujo et al., 2019). The outer mitochondrial membrane (OMM) controls metabolite exchange, apoptotic signaling, and protein import, and is also a platform for fusion/fission proteins. The inner mitochondrial membrane (IMM), which forms cristae, contains electron transport chain (ETC) complexes and ATP synthase; its lipid environment, including cardiolipin, is essential for respiratory efficiency and cristae stability. The intermembrane space (IMS) contains cytochrome c, whose release links mitochondrial injury to apoptosis. The matrix contains mitochondrial DNA (mtDNA), TCA cycle enzymes, calcium-buffering systems, and factors required for mtDNA transcription and translation (Lewis et al., 2016; Youle, 2019). In AD, damage to these compartments manifests as impaired respiration, altered membrane potential, mtDNA injury, disrupted cristae architecture, abnormal calcium handling, and vulnerability to apoptotic signaling (Soldatov et al., 2022; Babaylova et al., 2020; Singh et al., 2019; Jeje et al., 2024; Lackner, 2013; Lucero et al., 2019).

Because neurons have high energetic demands and long axonal processes, they depend on efficient mitochondrial trafficking, local ATP production, and rapid mitochondrial turnover (Yamashita et al., 2021; Zhang et al., 2022; Sun et al., 2016; Aranda-Anzaldo et al., 2024). Thus, relatively subtle mitochondrial defects can have disproportionate effects on synaptic transmission and plasticity. For AD-focused therapy, the most relevant mitochondrial features are therefore: (i) ETC function and ATP synthesis; (ii) redox balance and antioxidant capacity; (iii) calcium uptake and release; (iv) mitochondrial dynamics and axonal transport; (v) mitochondrial biogenesis and mitophagy; and (vi) communication with the endoplasmic reticulum and inflammatory signaling pathways (Luo et al., 2020; Maiuolo et al., 2021; Cojocaru et al., 2023).

### 2.1 Mitochondrial quality control and mitophagy

Mitochondrial quality control (MQC) maintains a functional mitochondrial population through coordinated biogenesis, fusion/fission, proteostasis, and mitophagy (Giordani et al., 2021). In neurons, MQC is especially important because damaged mitochondria must often be recognized in distal axons and transported or locally degraded before they compromise synaptic function (Chiang et al., 2021; Gilkerson et al., 2012; Tian et al., 2022). The PINK1/Parkin pathway is the best-characterized mitophagy mechanism: loss of mitochondrial membrane potential allows PINK1 stabilization on the OMM, recruitment of Parkin, ubiquitination of OMM proteins, and engagement of autophagy receptors. Receptor-mediated pathways involving BNIP3, NIX, FUNDC1, and related proteins provide additional routes for mitochondrial removal (Picca et al., 2021; Wang X.-L. et al., 2021). In AD models and patient-derived systems, impaired mitophagy is associated with accumulation of depolarized mitochondria, elevated ROS, defective Aβ/tau clearance, and neuroinflammation. However, the extent to which mitophagy impairment is causal versus compensatory remains context-dependent and should be interpreted according to model and disease stage (Hool, 2021; Marín-Aguilar et al., 2017).

For nanomedicine, MQC is therapeutically relevant because nanosystems may be designed either to remove damaged mitochondria, protect mitochondria from oxidative damage, or restore the signaling pathways that coordinate mitochondrial renewal (Geto et al., 2020; Mary et al., 2022; Li et al., 2020; Martinez-Vicente, 2017). These goals require careful dose control: excessive stimulation of autophagy or nonspecific mitochondrial disruption could aggravate neuronal stress rather than repair mitochondrial homeostasis (Pradeepkiran et al., 2025; Agarwal et al., 2022; Wang W. et al., 2024).

### 2.2 Mitochondrial biogenesis

Mitochondrial biogenesis depends on coordinated nuclear and mitochondrial gene expression. Peroxisome proliferator-activated receptor gamma coactivator-1 alpha (PGC-1α), nuclear respiratory factors (NRF-1/2), and mitochondrial transcription factor A (TFAM) are central regulators of respiratory chain component expression and mtDNA maintenance (Blesa et al., 2007; Inatomi et al., 2022). In AD, reductions in PGC-1α/NRF/TFAM signaling have been linked to reduced mitochondrial mass, impaired respiration, and weakened neuronal resilience. These changes may be driven by oxidative stress, Aβ/tau toxicity, inflammation, and age-related metabolic decline. Mitochondrial biogenesis is therefore a potential therapeutic target, but increasing mitochondrial number without ensuring quality control may simply expand a dysfunctional mitochondrial pool (Kim et al., 2018; Ye et al., 2023).

A balanced therapeutic approach should couple biogenesis with mitophagy and antioxidant defense. Nanocarriers delivering antioxidants, metabolic modulators, or nucleic acids may help restore this balance if they can achieve sufficient neuronal and mitochondrial exposure without causing off-target toxicity.

### 2.3 Mitochondrial dynamics and trafficking

Mitochondria continuously undergo fusion and fission. Fusion, mediated mainly by MFN1/2 and OPA1, supports content mixing, cristae maintenance, and stress resilience. Fission, mediated largely by Drp1 and its receptors, separates damaged mitochondrial segments for removal and supports mitochondrial distribution along axons (Tábara et al., 2024; Garcia et al., 2018). In AD, an imbalance toward excessive fission and reduced fusion has been reported in several experimental systems. Aβ and tau may influence this imbalance through ROS, calcium-dependent kinase signaling, abnormal Drp1 activation, and microtubule disruption. These effects can impair mitochondrial transport to synapses, reduce local ATP availability, and enhance vulnerability to apoptosis (Gilkerson et al., 2021; Michalska et al., 2018; Gargalionis et al., 2023).

## 3 Mitochondrial dysfunction in AD

Mitochondrial function is a critical determinant of cellular health, particularly within the high-metabolic environment of neurons. Although the nervous system constitutes only ∼2% of total body weight, it accounts for 20% of total oxygen consumption (Leung et al., 2021). Neurons exhibit exceptionally high energy demands, heavily relying on continuous ATP production to maintain ion gradients across cell membranes, support action potential generation and propagation, and enable neurotransmitter release (Wang H. et al., 2024; Wang S. et al., 2023). Mitochondria are thus indispensable components of neuronal function, intrinsically linking neuronal activity to mitochondrial energy production and oxygen supply. Moreover, the unique structural features of neurons further underscore the importance of mitochondria in these cells (Kamienieva et al., 2021; Ravindran and Gustafsson, 2025; Wang H. et al., 2024). Beyond their distribution throughout the cytoplasm, mitochondria are extensively localized within various neuronal projections-particularly along axons, some of which extend over 1 m in length. This intricate architecture necessitates finely tuned mitochondrial function to meet the distinct energy requirements of different cellular compartments (including axons, dendrites, and the soma) and requires efficient mitochondrial trafficking to ensure energy supply matches the heterogeneous demands of these structural elements (Aschrafi et al., 2016). Given this pivotal role, the nervous system is highly vulnerable to mitochondrial dysfunction. Indeed, most mitochondrial diseases manifest with neurological symptoms.

Conventional therapeutic strategies for neurological disorders often focus on symptom alleviation or slowing disease progression but frequently fail to address underlying mitochondrial dysfunction. Treatments such as neurotransmitter replacement, neuroprotective agents, and modulation of synaptic activity have limited efficacy as they do not correct impaired energy metabolism within neurons (Cunliffe et al., 2022). Furthermore, many neuroprotective drugs face challenges including poor BBB permeability and off-target effects, diminishing their clinical utility. The earliest indicators of many mitochondrial diseases typically involve neurological deficits, highlighting the strong correlation between mitochondrial dysfunction and the pathology of numerous neurological disorders. These limitations underscore the need for therapeutic approaches that can directly target and restore mitochondrial function, thus addressing the root causes of neuronal degeneration (Lukens and Williams, 2022; Lim et al., 2024).

**FIGURE 1:**
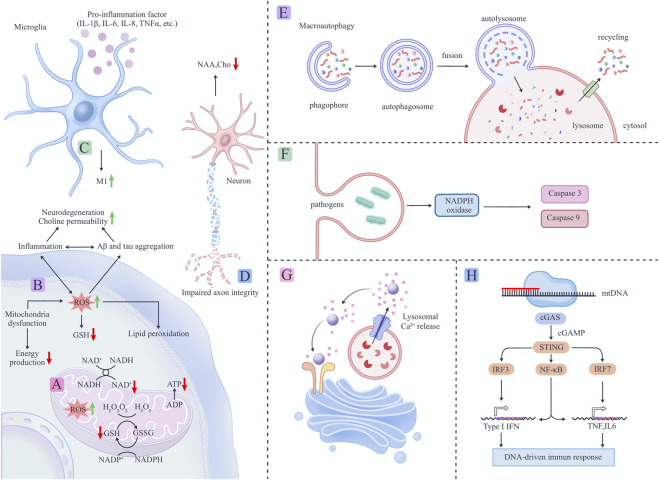
Mitochondrial damage mechanisms in AD. **(A)** Mitochondrial oxidative stress and ROS-mediated injury to lipids, proteins, and mtDNA. **(B)** Impaired mitophagy and accumulation of dysfunctional mitochondria. **(C)** Synaptic dysfunction caused by ATP deficiency and impaired mitochondrial trafficking. **(D)** Neuronal injury resulting from sustained metabolic and redox stress. **(E)** Mitophagy pathway, including recognition of depolarized mitochondria and lysosomal degradation. **(F)** ETC impairment and reduced ATP production, with Aβ-related mechanisms indicated as model-dependent. **(G)** Calcium dyshomeostasis, MAM dysfunction, mPTP opening, and apoptosis signaling. **(H)** Mitochondrial DAMP-driven neuroinflammatory amplification through microglial pathways.

As cellular “powerhouses,” mitochondrial dysfunction is a core driver of AD pathology. Impairments in energy metabolism-particularly defects in ATP synthesis-directly compromise neurons’ high energy requirements and exacerbate core AD pathological features (Aβ deposition, tau hyperphosphorylation, synaptic loss, neuronal death) through multiple pathways. The major mitochondrial damage mechanisms in AD, including oxidative stress, impaired mitophagy, synaptic energy failure, calcium dyshomeostasis, ETC dysfunction, apoptosis, and neuroinflammatory amplification (Figure 1).

### 3.1 Disorder of energy metabolism

#### 3.1.1 Mitochondrial electron transport chain damage: the core bottleneck in ATP synthesis

In AD patient brains (especially in vulnerable regions like the hippocampus and cortex), a significant reduction in the activity of ETC Complex IV is one of the most consistent and early findings (Cai et al., 2016). This directly hinders the terminal step of the electron transport chain, namely, the transfer of electrons from Cyt C to oxygen to generate water. This process is one of the key steps in establishing the proton gradient (H^+^ gradient). Soluble Aβ oligomers have been reported in selected experimental models to interact with mitochondrial respiratory components, including Complex IV, although the extent to which this mechanism dominates in sporadic human AD remains unresolved (Qian et al., 2025). It is reported that Aβ can specifically binds to the ABAD protein on the mitochondrial inner membrane. This binding not only inhibits the normal function of ABAD itself, more importantly, it triggers a series of cascade reactions, such as ROS burst and mitochondrial permeability transition pore (mPTP) opening (Hou et al., 2022). The Aβ-ABAD complex significantly increases the production of mitochondrial ROS (mainly superoxide anions). Excessive ROS further oxidatively damages ETC complexes (especially oxidation-sensitive Complex I and Complex IV), mtDNA, lipids, and proteins, forming a vicious cycle (e.g., the inflammatory and apoptotic processes regulated by the Caspase-3/9 family) (Cai et al., 2016). Aβ–ABAD interaction has been proposed as one mechanism linking Aβ accumulation to mitochondrial ROS generation and mPTP dysregulation, but its relative contribution likely depends on model system and disease stage (Mnatsakanyan et al., 2016). ROS and mPTP opening further damage the entire ETC function, exacerbating mitochondrial dysfunction, leading to ATP synthesis impairment. In summary, significantly reduced activity of ETC complexes (especially Complex IV) in AD brain tissue leads to decreased ATP generation. Aβ can directly inhibit Complex IV and exacerbate dysfunction by binding to the mitochondrial membrane protein ABAD (Youssef et al., 2025).

ATP is the foundation for almost all high-energy-consuming activities in neurons, including maintaining resting membrane potential, synaptic transmission (vesicle cycling, neurotransmitter synthesis and release), axonal transport, protein synthesis and degradation. ATP deficiency directly leads to low neuronal function and “idling” (Guedes-Dias and Holzbaur, 2019). Weakened function of ATP-driven ion pumps (such as Na^+^/K^+^-ATPase) leads to intracellular Na^+^ accumulation, K^+^ loss, membrane depolarization, damaging neuronal excitability and signal transmission. Mitochondria are important calcium buffers (Zhang et al., 2022). ETC dysfunction and reduced MMP weaken the ability of mitochondria to uptake calcium. Simultaneously, the function of ATP-dependent endoplasmic reticulum calcium pumps is also impaired, leading to abnormally elevated cytosolic calcium concentration and calcium overload (de Araujo et al., 2019; Yamashita et al., 2021). Calcium overload activates multiple calcium-dependent proteases (such as Calpain), phosphatases (such as Calcineurin), and kinases. These enzymes hyperphosphorylate tau protein, promote neurofibrillary tangle formation, thereby activating pro-apoptotic pathways, which then further damage mitochondrial function (Shen et al., 2017). On the other hand, synapses are “hotspots” of energy consumption. ATP insufficiency severely affects neurotransmitter release at presynaptic terminals and the function of postsynaptic receptors, leading to weakened long-term potentiation and enhanced long-term depression, manifesting as learning and memory impairments. Ultimately, energy-depleted synapses will be pruned or degenerate (Hollnagel et al., 2020).

#### 3.1.2 Abnormal glucose metabolism: fuel supply and processing disorders

In AD patient brains, decreased glucose utilization (cerebral hypometabolism) may be associated with inhibition of mitochondrial pyruvate dehydrogenase (PDH) activity and lactate accumulation (Lukens and Williams, 2022). Years or even decades before the onset of obvious clinical symptoms in AD patients, FDG-PET studies show that cerebral glucose hypometabolism can precede overt dementia, making it an important biomarker of early AD-related dysfunction (Mente et al., 2017). PDH inhibition may reduce the conversion of pyruvate to acetyl-CoA and thereby weaken TCA cycle flux and oxidative phosphorylation. Nevertheless, PDH dysfunction is only one component of AD metabolic impairment (Singh and Singh, 2021). Central insulin resistance may reduce PI3K-AKT signaling and impair neuronal glucose utilization; altered GLUT1 at the BBB and GLUT3 in neurons may limit glucose delivery and uptake; and disruption of astrocyte-neuron metabolic coupling may reduce lactate shuttling to neurons (Velazquez et al., 2017). Microvascular dysfunction and chronic inflammation can further restrict substrate supply and mitochondrial oxidation.

These interacting mechanisms may explain why targeting only one metabolic enzyme has generally been insufficient. Therapeutic strategies should therefore aim to improve substrate availability, mitochondrial oxidation, redox balance, and glial-neuronal metabolic cooperation. Nanomedicine may contribute by delivering metabolic modulators, antioxidants, or gene regulators to defined brain cell types, but this requires careful validation in models that reproduce human AD metabolic heterogeneity.

#### 3.1.3 Obstacle in the utilization of alternative energy substrates: failure of compensatory mechanisms

Weakened compensatory capacity of ketone body metabolism will also exacerbate the neuronal energy crisis. When glucose supply is insufficient or utilization is impaired (such as during starvation, diabetes), ketone bodies (mainly β-hydroxybutyrate, BHB) are important alternative energy sources for the brain (Bertero et al., 2018). Ketone bodies are produced in the liver, cross the BBB, and are taken up by neurons and astrocytes. Inside mitochondria, BHB is converted into acetoacetate, which is then converted by succinyl-CoA transferase (SCOT) into acetoacetyl-CoA, ultimately entering the TCA cycle for oxidative energy production (Stagg et al., 2021). Ketone body metabolism is highly efficient and produces less ROS than glucose metabolism. As mentioned earlier, the activity of the key ketone body utilization enzyme SCOT is significantly reduced in AD brains (Balestra et al., 2024). This means that even if ketone bodies enter neurons, the ability to effectively convert them into acetoacetyl-CoA for entry into the TCA cycle is weakened. Additionally, the overall dysfunction of the TCA cycle and ETC also limits the efficiency of ATP production through ketone body oxidation. When the “main road” of glucose metabolism is severely obstructed (cerebral hypometabolism + PDH inhibition), the brain instinctively needs to rely on the “auxiliary road” (ketone bodies) to maintain energy supply (Brahma et al., 2021). However, this critical compensatory pathway (ketone body utilization) also fails in AD (Shippy et al., 2024) (SCOT activity reduction, etc.). This makes neurons more vulnerable when facing glucose metabolism disorders, completely sinking into a deep and unrelieved energy crisis, accelerating functional failure and death.

Overall, ATP synthesis defects caused by mitochondrial energy metabolism disorders (ETC damage (Singh et al., 2019), glycolysis-TCA cycle connection impairment (Corbet et al., 2016), alternative energy utilization failure (Babaylova et al., 2020)) are the core driving force for neuronal functional decline and death in AD: 1) Formation of a vicious cycle: AD pathological proteins such as Aβ and p-tau directly or indirectly (through ROS, calcium overload) damage mitochondria (especially ETC Complex IV and PDH), leading to reduced ATP production (Wang B. et al., 2021). ATP deficiency in turn weakens the neuron’s ability to clear Aβ (e.g., affecting proteasome, autophagy) and prevent tau hyperphosphorylation (affecting kinase/phosphatase balance), further exacerbating AD pathology, forming a self-reinforcing vicious cycle; 2) Comprehensive collapse of neuronal function: ATP scarcity directly affects the most basic functions of neurons-maintaining membrane potential, ion homeostasis, synaptic transmission (physiological basis of learning and memory), axonal transport (transport of nutrients and signaling substances), protein homeostasis maintenance. This directly manifests as cognitive dysfunction (Wang H. et al., 2024); 3) Activation of cell death pathways: Persistent ATP deficiency, severe ROS bursts, calcium overload, and mPTP opening ultimately push neurons toward apoptotic or necrotic death, leading to irreversible brain atrophy (Hou et al., 2022; Mnatsakanyan et al., 2016).

### 3.2 Oxidative stress and free radical damage

In the pathological process of AD, mitochondrial oxidative stress and free radical damage synergistically drive neurodegeneration through a triple mechanism (Nolfi-Donegan et al., 2020; Christofides et al., 2021): 1) The antioxidant defense system collapses comprehensively: key antioxidants in the brains of AD patients are significantly reduced, manifested as a 30%–50% decrease in glutathione (GSH) levels (particularly in the hippocampus and cortex) and a decline in superoxide dismutase (SOD) activity (Xie et al., 2022). GSH depletion results from excessive ROS consumption, Aβ inhibition of γ-glutamylcysteine ligase (GCL) synthesis, and mitochondrial membrane transport dysfunction; while SOD2 (mitochondrial Mn-SOD) is inactivated due to direct binding by Aβ, nitration modification, and mtDNA damage, leading to the loss of superoxide anion (·O_2_^−^) scavenging capacity (Thangamani et al., 2017); 2) Mitochondrial ETC dysfunction triggers explosive ROS generation: Aβ oligomers inhibit complex I (binding to the NDUFS1 subunit), III (damaging cytochrome b), and IV (directly blocking COX activity), causing electron leakage and the accumulation of ·O_2_^−^ and hydrogen peroxide (H_2_O_2_) within mitochondria (Christofides et al., 2021; Babaylova et al., 2020). These reactive oxygen species attack mtDNA, causing deletion mutations (forming a “ROS-mtDNA-ETC decline” cycle), triggering lipid peroxidation to produce 4-hydroxynonenal (4-HNE) and malondialdehyde (MDA)—the former diffuses into the cytoplasm to modify tau protein and promote phosphorylation, while the latter cross-links membrane proteins and disrupts mitochondrial integrity (Tian et al., 2018); 3) Aβ and ROS form a self-reinforcing vicious cycle: extracellular Aβ activates microglial NOX2 and neuronal RAGE receptors, inducing cytoplasmic ·O_2_^−^ bursts; simultaneously, Aβ invades mitochondria to inhibit SOD2, consume GSH, and bind to ABAD to inhibit complex IV, further amplifying mitochondrial ROS (Shitaw et al., 2025). The accumulated ROS significantly promote Aβ production and hinder its clearance by upregulating BACE1 expression (NF-κB/JNK pathway) and oxidizing inactivating insulin-degrading enzyme, constituting a continuously amplifying pathological axis (Chen E. et al., 2024). This triangular cycle (antioxidant collapse-ETC leakage-Aβ amplification) ultimately leads to energy failure, exacerbated tau pathology, and neuronal death, becoming the core driving force of AD neurodegeneration (Figures 1A,B).

### 3.3 Calcium homeostasis imbalance and apoptosis activation

Mitochondria and the endoplasmic reticulum (ER) cooperate to regulate intracellular calcium. MAMs are specialized contact sites that coordinate calcium transfer, lipid metabolism, autophagy initiation, and inflammatory signaling (de Araujo et al., 2019; Maiuolo et al., 2021). In AD, altered MAM function has been implicated in excessive ER-to-mitochondria calcium transfer, increased mitochondrial calcium load, mPTP opening, and activation of apoptotic pathways (Zhang et al., 2022). Presenilin mutations and APP-processing components have also been linked to MAM biology, suggesting a connection between familial AD mechanisms and organelle communication.

When mitochondrial calcium buffering fails, elevated mitochondrial calcium can impair respiration, increase ROS, lower the threshold for mPTP opening, and promote cytochrome c release. The released cytochrome c forms the apoptosome with Apaf-1 and activates caspase-9 and caspase-3, leading to apoptosis (Hou et al., 2022).

**FIGURE 2:**
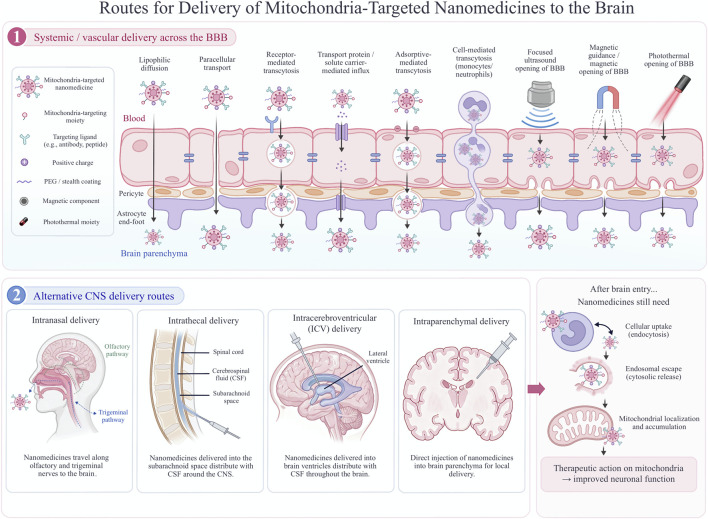
BBB traversal and alternative CNS delivery routes for mitochondria-targeted nanomedicines in AD. This schematic summarizes the major delivery routes by which mitochondria-targeted nanomedicines may reach the brain in AD. Systemic vascular delivery across the BBB may occur through lipophilic diffusion, paracellular transport, receptor-mediated transcytosis, transport protein-/solute carrier-mediated influx, adsorptive-mediated transcytosis, and cell-mediated transcytosis. Physical BBB-modulation strategies, including focused ultrasound, magnetic guidance, and photothermal opening, may transiently enhance endothelial permeability and facilitate nanomedicine accumulation in the brain. In addition to intravenous administration, alternative CNS delivery routes such as intranasal, intrathecal, intracerebroventricular, and intraparenchymal administration may partially bypass or directly access CNS compartments. Each route has distinct advantages and limitations in terms of invasiveness, dose capacity, distribution efficiency, safety, and translational feasibility. After brain entry, nanomedicines must still achieve cellular uptake and mitochondrial localization, highlighting the dual-targeting requirement of AD mitochondrial nanotherapy.

The uncontrolled opening of the mPTP triggers a triple cascade reaction (Mnatsakanyan et al., 2016): 1) Calcium ions in the mitochondrial matrix flow back to the cytoplasm, exacerbating intracellular calcium overload (Figure 1G); 2) Mitochondrial swelling and outer membrane rupture, releasing the apoptotic initiator protein Cyt C; 3) ATP synthesis is completely interrupted, leading to an irreversible energy crisis. The Cyt C released into the cytoplasm binds with Apaf-1 to form an apoptosome (Ma et al., 2021), activating caspase-9 and cascading to effect caspase-3, ultimately triggering irreversible neuronal apoptosis (Figures 1D,F). This process forms a positive feedback loop with Aβ deposition and tau phosphorylation, jointly promoting the progressive loss of hippocampal and cortical neurons and brain atrophy in AD patients (Feng et al., 2024). These pathways are well established in cell biology, but their precise timing and dominance in human AD remain difficult to determine.

### 3.4 Mitochondrial dynamics imbalance and abnormality of the quality control system

During the progression of AD, the imbalance in mitochondrial dynamics and the dysfunction of the quality control system jointly lead to the accumulation of damaged mitochondria and their functional collapse. The imbalance in the mitochondrial fusion-fission dynamics is manifested as enhanced phosphorylation of the fission protein DRP1 at Ser616, which is induced by calcium overload and excessive ROS activation, alongside a significant downregulation of the fusion proteins MFN2 (mediating mitochondrial outer membrane fusion) and OPA1 (regulating inner membrane fusion). This imbalance results in fragmentation of the mitochondrial network, producing short and dispersed mitochondria with reduced membrane potential (MMP) and lost ATP synthesis capacity (Dai et al., 2020). Although fragmented mitochondria should activate the PINK1/Parkin pathway via PINK1 stabilization and Parkin recruitment on the mitochondrial outer membrane, this process is directly inhibited in AD. Aβ oligomers suppress the E3 ubiquitin ligase activity of Parkin, while oxidative stress impairs PINK1 stability, ultimately preventing autophagosomes from effectively encapsulating and clearing mitochondria containing damaged mtDNA (Du et al., 2022). Ultimately, mitochondrial biogenesis is continuously inhibited due to ROS and Aβ released from fragmented mitochondria, which inhibit the transcriptional co-activator PGC-1α. This inhibition blocks the downstream NRF-1/TFAM signaling axis, reduces the synthesis of nuclear-encoded mitochondrial components, and decreases the new mitochondrial generation rate by more than 40% (Tian et al., 2018). This “hyperfission-hypofusion-autophagy failure-biogenesis inhibition” quadruple failure forms an irreversible break in the mitochondrial renewal cycle, becoming a key driver of the progressive degeneration of AD neurons (Figure 1E).

### 3.5 The mitochondrial toxicity of Aβ and tau proteins

Aβ and tau can converge on mitochondria through partially distinct mechanisms. Soluble Aβ species have been detected in mitochondria in several experimental systems and may affect respiratory complexes, ABAD, mitochondrial import machinery, and mPTP regulation. tau pathology, by contrast, is strongly linked to microtubule instability, axonal transport defects, altered mitochondrial distribution, and Drp1-related fragmentation. Both proteins may increase oxidative stress and impair synaptic mitochondrial supply (Gilkerson et al., 2021; Gargalionis et al., 2023).

Aβ and tau protein jointly disrupt mitochondrial function through a dual pathway: 1) Aβ directly deposits in mitochondria: Soluble Aβ oligomers invade the mitochondrial matrix through the mitochondrial outer membrane translocation enzyme (TOM) and inner membrane translocation enzyme (TIM) complexes, targeting and binding to the key component of the ETC-the inhibitory complex IV activity (blocking the transfer of electrons to oxygen), and inducing the collapse of MMP, resulting in a sudden drop of ATP synthesis. At the same time, Aβ-mitochondrial contact triggers the explosive production of ROS, forming a “Aβ retention-ETC inhibition-energy exhaustion” self-destructive cycle (Dou and Tan, 2023); 2) Excessive phosphorylated tau protein disrupts mitochondrial transport: Pathological tau (p-tau, phosphorylated at Ser396/404 sites) abnormally aggregates in axons, competitively binding to the motor domain of dynamin, blocking its coupling with the mitochondrial outer membrane protein Miro/TRAK complex, causing a retrograde transport disorder of mitochondria along microtubules towards the synaptic terminal (Feng et al., 2024). The accumulation of mitochondria in neuronal cell bodies induces local oxidative stress. Concurrently, a deficiency of mitochondria in presynaptic terminals leads to insufficient ATP supply for synaptic vesicle cycling and neurotransmitter release, which accelerates synaptic atrophy (Lukens and Williams, 2022; Cuyvers and Sleegers, 2016). These two pathologies create a synergistic dual toxicity effect, whereby endogenous mitochondrial damage induced by Aβ and mitochondrial spatial distribution disorder caused by p-tau jointly weaken the neuronal energy metabolism network, forming the structural basis for cognitive decline in AD (Da et al., 2024).

These mechanisms are biologically plausible and supported by multiple preclinical studies, but the abundance, localization, and molecular form of Aβ/tau differ across models and human disease stages, and mitochondrial injury may also arise from aging, vascular dysfunction, inflammation, and metabolic stress (Figure 1C). Thus, mitochondria should be viewed as a convergence point where Aβ/tau-dependent and Aβ/tau-independent mechanisms interact.

### 3.6 The amplifying effect of neuroinflammation

Neuroinflammation, through a self-reinforcing pathological cascade reaction, becomes a key accelerator in amplifying mitochondrial dysfunction in AD. This vicious cycle begins with the release of molecular alarms from damaged mitochondria. Specifically, when mitochondria suffer severe oxidative damage or fragmentation, the exposed cardiolipin (a phospholipid normally located in the inner membrane) is directly recognized by the NLRP3 receptor as a “eat me” signal (Pizzuto and Pelegrin, 2020). Simultaneously, the leaked fragmented mtDNA, which contains unmethylated CpG motifs, binds to Toll-like receptor 9 (TLR9) in the endosomal compartments of microglia, thereby activating the downstream MyD88/NF-κB signaling axis and initiating the transcription of pro-inflammatory genes. Meanwhile, ATP leaked into the extracellular space binds to the purinergic receptor P2X7, triggering K^+^ efflux and a sudden increase in intracellular ROS. These three types of DAMPs jointly induce the assembly of the NLRP3 inflammasome (containing ASC adaptor protein and pro-caspase-1), leading to the autocleavage of caspase-1, which then catalyzes the maturation of pro-interleukin-1β (pro-IL-1β) and IL-18 into their active forms (Tian et al., 2018). The resulting neuroinflammatory storm is bidirectionally destructive. Activated M1-type microglia secrete large amounts of IL-1β, TNF-α, and IL-6. Among these, TNF-α binds to the neuronal TNFR1 receptor, activating the RIPK1/ROS pathway and directly inhibiting the activity of the electron transport chain complex I (NADH dehydrogenase). This inhibition results in a 200% increase in electron leakage and an explosive generation of mitochondrial ·O_2_^−^. Concurrently, IL-1β downregulates the expression of γ-glutamylcysteine ligase C (GCLC), which is the rate-limiting enzyme for the synthesis of GSH. This suppression causes a 40% decrease in neuronal GSH levels and a consequent collapse of the antioxidant defense system (Tsviklist et al., 2022). Even more seriously, these inflammatory mediators further deteriorate mitochondrial integrity. For instance, ROS oxidatively modifies the Cys644 site of the DRP1 protein, enhancing its GTPase activity and accelerating mitochondrial division. Meanwhile, TNF-α induces the overexpression of the periplasmin protein D (CypD) in the mitochondrial mPTP, reducing the threshold for pore opening (Leung et al., 2021; Tsviklist et al., 2022). Ultimately, fragmented mitochondria continuously release DAMPs, forming a self-perpetuating cycle that progresses from mitochondrial damage to TLR9/NLRP3 inflammation activation, then to a TNF-α/IL-1β explosion, resulting in ETC inhibition/antioxidant failure, and leading to secondary mitochondrial disintegration (Chinenye et al., 2024). This cycle not only independently drives neuronal death but also engages in cross-dialogue with Aβ/tau pathology. For example, IL-1β upregulates the expression of β-secretase to promote Aβ production, while oxidative mtDNA can activate the cGAS-STING pathway to induce excessive phosphorylation of tau. These interactions jointly constitute an explosive progression network for AD neurodegeneration (Figure 1H).

### 3.7 Integration with the mitochondrial cascade hypothesis

The mitochondrial cascade hypothesis provides an integrative framework in which baseline mitochondrial function, inherited mitochondrial variation, aging, and environmental stress determine neuronal resilience (Yang L. et al., 2020). Mitochondrial deficits can promote Aβ accumulation and tau phosphorylation by impairing proteostasis, autophagy, kinase/phosphatase balance, and redox homeostasis. Aβ and tau can then further impair mitochondrial respiration, trafficking, and quality control. This reciprocal relationship helps explain why therapies directed only at extracellular Aβ may not fully reverse established neuronal dysfunction (Kamienieva et al., 2021; Ravindran and Gustafsson, 2025; Wang H. et al., 2024).

For therapeutic design, the implication is that mitochondrial intervention may be most useful when applied early or when combined with approaches that reduce Aβ/tau burden and neuroinflammation. Mitochondria-targeted nanomedicine is attractive in this context because a single platform may combine BBB penetration, neuronal targeting, mitochondrial localization, antioxidant activity, gene regulation, and anti-inflammatory effects (Wang S. et al., 2023; Nolfi-Donegan et al., 2020).

## 4 Dual targeting strategies: crossing the BBB and reaching neuronal mitochondria

### 4.1 Systemic/vascular delivery relies on several mechanisms

Efficient delivery of therapeutic agents to the mitochondria within neurons is the primary challenge in targeted treatment of AD (Figure 2). The core issue lies in how to overcome the strictly regulated BBB. The BBB is composed of endothelial cells connected by tight junctions, and is surrounded by the foot processes of astrocytes on the periphery. This significantly restricts the passive diffusion of substances from the blood to the brain.

To overcome this barrier, researchers have designed various nanoparticles and utilized one or more of the following mechanisms to achieve trans-BBB transport (Chiang et al., 2021). 1) Lipophilic diffusion: Design small-sized nanoparticles with appropriate lipid solubility, enabling them to simulate small molecule drugs and undergo passive diffusion through the lipid bilayer of the cell membrane. This is the simplest and most direct method, but it imposes strict requirements on the physicochemical properties of the nanomaterials (Michalska et al., 2018). 2) Receptor-mediated transcytosis: this is currently the most promising active targeting strategy. By modifying specific ligands (such as transferrin, lactoferrin, antibodies or peptide segments targeting low-density lipoprotein receptors, etc.) on the surface of nanoparticles, they can bind to the specific receptors highly expressed on the BBB endothelial cells, and then be endocytosed and transported to the brain parenchyma. This method is highly efficient and has strong targeting ability (Inatomi et al., 2022). 3) Adsorptive-mediated transcytosis: this process utilizes positively charged nanoparticles (such as those modified with cell transmembrane peptides or cationic albumin) to form electrostatic adsorption with the negatively charged BBB endothelial cell membrane, inducing membrane invagination and completing the transport. This method is relatively simple to prepare, but has slightly lower selectivity (Balestra et al., 2024). 4) Transport protein-mediated/Solute carrier protein influx: Design nanoparticles to mimic the structure of endogenous nutrients (such as glucose, amino acids), and use the abundant natural transport proteins on the BBB (such as GLUT1, LAT1, etc.) to “carry” them into the brain. This is a biomimetic strategy with good biocompatibility (Chang et al., 2018). 5) Cell-mediated transcytosis: Nanoparticles are loaded into immune cells (such as macrophages and neutrophils), and by leveraging the inherent ability of these cells to cross the BBB, they are delivered into the brain as a “Trojan horse.” Subsequently, the nanoparticles are released to exert therapeutic effects (Martinez-Vicente, 2017). 6) Temporary opening of the BBB by physical methods: Compared to biochemical strategies, physical methods offer a controllable and non-invasive way to open the BBB (Liu B.-H. et al., 2024). 7) Ultrasound opening of BBB: After intravenous injection of microbubbles, focused ultrasound is applied to a specific brain region. The oscillation of the microbubbles can temporarily and reversibly relax the tight junctions, thereby significantly enhancing the permeability of the nanoparticles (Lucero et al., 2019). 8) Magnetic activation of BBB: By applying an external magnetic field to the targeted magnetic nanoparticles that are encapsulated within the BBB, mechanical force is used to disturb the endothelial cells, thereby increasing their permeability (Corbet et al., 2016). 9) Photothermal activation of BBB: Utilizing nanomaterials with photothermal properties (such as gold nanorods, carbon nanotubes), local heat is generated under near-infrared laser irradiation, causing a thermal effect that can reversibly open the tight junctions (Zhang et al., 2022). In conclusion, choosing the appropriate transmembrane mechanism is a prerequisite for designing targeted mitochondrial nanomedicines (Tsviklist et al., 2022).

Mitochondrial targeting shows promise in the treatment of AD. Gao et al. developed a red blood cell membrane-based nanodrug delivery system, leveraging the high expression of the CD47 protein on the red blood cell surface (Du et al., 2022). CD47 plays a crucial role in evading phagocytosis by the reticuloendothelial system, thereby prolonging the circulation time of nanoparticles and reducing immune recognition. Furthermore, the red blood membrane surface was modified with the positron emission tomography (PET) imaging agent T807, which enables efficient crossing of the BBB and specific binding to neuronal cells. This system was further refined for mitochondrial targeting through the incorporation of TPP, encapsulating curcumin within the core-shell structure formed by the cell membrane. This strategy significantly improved cognitive and behavioral functions in AD model mice, demonstrating the therapeutic potential of a targeted delivery system combining the advantages of the cell membrane and the nanoparticle core (Chan, 2020; Skawratananond et al., 2025; McGill Percy et al., 2025; Pahal et al., 2025). The pathogenesis of AD is closely related to the aggregation of Aβ protein. In the early stages of AD, microglia-the brain’s resident immune cells—are activated in response to neuronal cell death, phagocytosing dead cells and presenting major histocompatibility complex (MHC) antigens. However, chronic overactivation of microglia leads to a vicious cycle of Aβ accumulation and neuroinflammation. Regulating microglial autophagy offers a potential strategy to modulate the brain’s inflammatory microenvironment, thereby alleviating oxidative stress and exerting neuroprotective effects (Youle, 2019; Soldatov et al., 2022; Babaylova et al., 2020; Singh et al., 2019). To target mitochondria and exert neuroprotective effects in the context of AD, Zhong et al. developed a Prussian blue/polyamidoamine (PAMAM) dendrimer/angiopep-2 nanosystem. Prussian blue, an FDA-approved antioxidant, was selected for its excellent safety profile and potent antioxidant capacity. PAMAM dendrimers are classic drug delivery carriers, functionalized with angiopep-2, a ligand for the low-density lipoprotein receptor-related protein-1, which is highly expressed on the surface of brain capillary endothelial cells. This targeting strategy facilitates efficient BBB penetration and targeted delivery to neuronal mitochondria, where it can modulate oxidative stress and provide neuroprotection (Wang S. et al., 2023; Nolfi-Donegan et al., 2020; Christofides et al., 2021; Corbet et al., 2016).

### 4.2 Alternative CNS delivery routes

Intranasal administration can partially bypass the BBB through olfactory and trigeminal pathways and may reduce systemic exposure, although dose capacity, mucociliary clearance, enzymatic degradation, and inter-individual variability remain limitations. Intrathecal and intracerebroventricular administration can deliver agents directly into cerebrospinal fluid, but they are invasive and may still provide limited penetration into deep brain parenchyma. Intraparenchymal delivery can achieve local exposure but is unsuitable for diffuse AD pathology except in specialized experimental settings. Therefore, the delivery routes are not limited to intravenous delivery; systemic, intranasal, and direct CNS routes each have distinct feasibility and safety profiles (Chinenye et al., 2024).

After brain entry, mitochondrial localization is commonly achieved by exploiting the negative mitochondrial membrane potential using lipophilic cations such as triphenylphosphonium (TPP), by using mitochondria-penetrating peptides, or by designing stimuli-responsive carriers that release cargo in ROS-rich or acidic intracellular environments. These approaches improve subcellular targeting but may also accumulate in non-neuronal mitochondria, disturb mitochondrial membrane potential, or produce dose-dependent toxicity. Thus, effective AD nanotherapy requires optimization of both brain accumulation and mitochondrial specificity (Lukens and Williams, 2022; Ravindran and Gustafsson, 2025; Wang H. et al., 2024; Wang S. et al., 2023; Nolfi-Donegan et al., 2020; Christofides et al., 2021; Corbet et al., 2016).

In summary, the strategic targeting of mitochondria in degenerative neurological diseases holds considerable promise. By leveraging the unique properties of nanoparticles, functionalizing them with targeting ligands, and encapsulating therapeutic agents, researchers are developing systems capable of crossing the BBB, reaching mitochondria within affected neurons, and alleviating the underlying mitochondrial dysfunction contributing to disease progression. As our understanding of mitochondrial biology and its role in neurodegenerative diseases deepens, these innovative approaches may yield new and effective therapeutic strategies.

## 5 Nanomedicine strategies for mitochondrial targeting in Alzheimer’s disease

**FIGURE 3:**
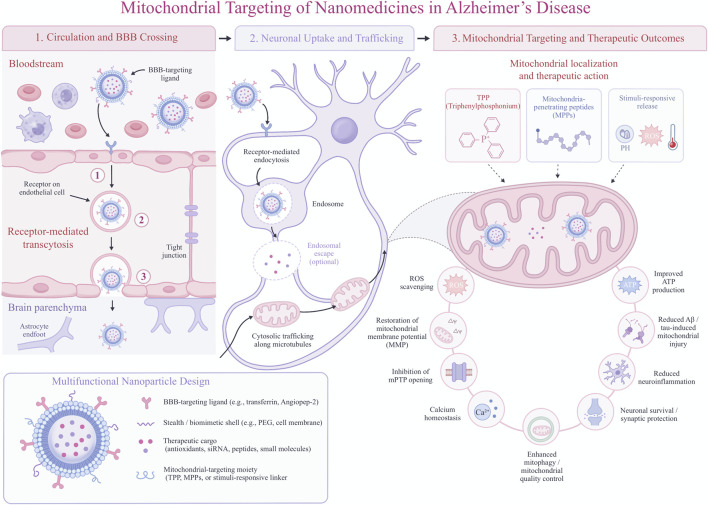
Mitochondrial targeting and major therapeutic mechanisms of nanomedicines in AD. This schematic illustrates the intracellular targeting sequence and major mitochondrial therapeutic mechanisms of nanomedicines in AD. After entering the brain parenchyma, nanomedicines must be internalized by neurons or other relevant brain cells, escape from endosomal compartments when required, and traffic toward mitochondria. Mitochondrial localization can be facilitated by several representative strategies, including lipophilic cations such as TPP, mitochondria-penetrating peptides, and stimuli-responsive systems that release cargo in response to pathological cues such as ROS, acidic pH, or mitochondrial membrane potential changes. Once localized to mitochondria, these nanomedicines may restore mitochondrial homeostasis by scavenging mitochondrial ROS, restoring mitochondrial membrane potential, inhibiting mPTP opening, rebalancing calcium homeostasis, improving ATP production, enhancing mitophagy and mitochondrial quality control, and reducing Aβ- and tau-induced mitochondrial injury. These effects may subsequently attenuate neuroinflammation, protect synapses and neurons, and improve AD-related functional outcomes. This figure emphasizes shared therapeutic mechanisms rather than individual nanosystem-specific details.

As an emerging field of AD treatment, mitochondria-targeted nanomedicine has made breakthroughs in recent years through multidisciplinary integration. The mitochondrial targeted drug delivery strategy should not only consider the physicochemical properties of the drug, but also consider the drug preparation and biological barriers to achieve intracellular targeting, so as to maximize the efficacy of the drug and minimize the side effects (Li et al., 2024). The efficacy of traditional drug therapy is limited by the blockage of the BBB and the low efficiency of mitochondrial targeting (Huang Y. et al., 2024). Nanomedicine overcomes the limitations of low bioavailability and non-specific delivery of drug molecules through precise carrier design and functional modification, and achieves efficient targeted intervention of neuronal mitochondria (Yadav et al., 2022) (Figure 3).

### 5.1 Dual-target delivery nanosystems for BBB traversal and mitochondrial localization

The first and still most established strategy is to engineer nanocarriers that solve the serial delivery problem in a stepwise manner: prolonged circulation, BBB penetration, neuronal uptake, and mitochondrial docking (Wang Y. et al., 2025). In this category, the nanoparticle primarily functions as a delivery vehicle, while the therapeutic payload may be an antioxidant, a bioenergetic modulator, or an anti-inflammatory agent. For BBB-oriented formulations, the design window is usually narrower than the broad 1–1,000 nm definition of “nanoparticles”; in practice, many CNS-directed systems are engineered in the 10–100 nm range, often around 20–200 nm, because particle size, shape, surface charge, and ligand presentation jointly affect endothelial interaction and transcytosis efficiency (Wang Y. et al., 2025).

Organic nanocarriers remain the dominant platform in this class because they offer flexible surface chemistry, biocompatibility, and scalable formulation methods. Representative examples include liposomes, polymeric nanoparticles, dendrimers, nanostructured lipid carriers, and micelles (Gong et al., 2021). In AD models, these systems are frequently decorated with BBB-targeting ligands such as RVG29, T807, lactoferrin, or angiopep-2, and then further modified with mitochondrial ligands such as TPP or SS-31 (Dal Magro et al., 2017). Gao et al. (2020) reported red-blood-cell-membrane-camouflaged human serum albumin nanoparticles co-modified with T807 and TPP to deliver curcumin to neuronal mitochondria, thereby mitigating mitochondrial oxidative stress and neuronal death in AD mice. Han et al. (2020) used a similar dual-target logic in resveratrol-loaded RBC-membrane-coated nanostructured lipid carriers bearing RVG29 and TPP, and later extended the concept to macrophage-membrane-coated solid lipid nanoparticles loaded with genistein, improving immune evasion and circulation persistence while maintaining neuronal mitochondrial targeting.

These studies illustrate why dual-target organic systems remain attractive: they are modular, drug-agnostic, and generally more adaptable than single-function nanoparticles. However, they also reveal several recurring limitations (Han et al., 2021). First, each additional targeting element increases synthetic complexity and batch-to-batch variability (Rajkumar et al., 2023). Second, electrostatic mitochondrial targeting through TPP, while effective, may not be completely selective for diseased mitochondria and can potentially perturb membrane potential at high local concentrations (Marrache and Dhar, 2012). Third, many studies rely on short-term murine efficacy readouts, whereas long-term biodistribution, metabolite profiling, and repeated-dose neurotoxicity remain insufficiently characterized (Sharma et al., 2018). Therefore, dual-target delivery systems are pharmacologically rational, but their translational value will depend on simplification of composition and rigorous *in vivo* validation rather than continued escalation of design complexity.

**FIGURE 4:**
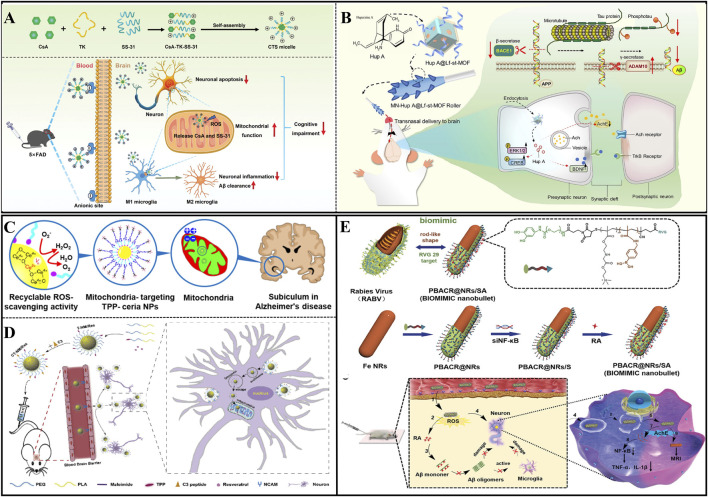
Representative nanomedicine strategies for mitochondrial-targeted therapy in Alzheimer’s disease. **(A, B)** Dual-targeting nanomedicine strategies. **(A)** Schematic illustration of ROS-responsive CsA-TK-SS-31 (CTS) micelles designed for sequential BBB penetration and mitochondrial targeting. After crossing the BBB, CTS micelles accumulate in neurons and microglia, where the thioketal linker is cleaved in the high-ROS mitochondrial microenvironment, releasing cyclosporin A (CsA) and SS-31 to restore mitochondrial function, reduce neuronal apoptosis and neuroinflammation, promote Aβ clearance, and improve cognitive impairment in AD models (Qian et al., 2025). Copyright 2025, Wiley-VCH GmbH. **(B)** Schematic illustration of a lactoferrin-functionalized cyclodextrin-based metal-organic framework nanocarrier for brain-targeted delivery of huperzine A. This platform integrates intranasal/brain-oriented delivery, BBB targeting, and synaptic/neuronal protection, thereby reducing oxidative injury and improving AD-related pathological processes, including Aβ/tau-associated synaptic dysfunction (Ruan et al., 2024). Copyright 2024, Elsevier. **(C, D)** Antioxidant and redox-regulating mitochondrial nanomedicine strategies. **(C)** Mitochondria-targeted TPP-ceria nanoparticles act as recyclable ROS-scavenging nanozymes. Through triphenylphosphonium (TPP)-mediated mitochondrial accumulation, these nanoparticles reduce mitochondrial oxidative stress and restore mitochondrial function in AD-associated brain regions (Kwon et al., 2016). Copyright 2016, American Chemical Society. **(D)** Schematic illustration of neuron- and mitochondria-targeted antioxidant nanocarriers co-modified with neural cell adhesion molecule (NCAM)-mimetic peptide C3 and TPP. This dual-ligand strategy enhances neuronal uptake and mitochondrial localization, enabling antioxidant cargo delivery to damaged neuronal mitochondria and reversal of mitochondrial dysfunction (Yang P. et al., 2020). Copyright 2020, Elsevier. **(E)** Biomimetic membrane-based targeting strategy. Inspired by rabies virus neurotropism, the biomimetic nanobullet PBACR@NRs/SA integrates BBB penetration, neuronal targeting, ROS-responsive retinoic acid release, Aβ aggregation inhibition, endosomal/lysosomal escape, acetylcholinesterase-responsive siNF-κB release, and MRI capability. This biomimetic platform reduces NF-κB-mediated inflammatory signaling and pro-inflammatory cytokine production, thereby combining neuronal targeting, anti-amyloid activity, immunomodulation, and diagnostic imaging in AD therapy (Wu et al., 2013). Copyright 2024, Wiley-VCH GmbH.

Zhou et al. designed a micelle, CsA-TK-SS-31 (CTS), which targets microglia and neurons. It blocks the progression of AD by simultaneously alleviating mitochondrial dysfunction (Dal Magro et al., 2017). SS-31 facilitates mitochondrial targeting, whereas the nanocarrier design supports BBB penetration and cellular uptake. In the mitochondria damaged by microglia and neurons, (thiosemicarbazone, CsA and SS-31 between them via TK) is destroyed. CsA and SS-31 are released when they consume reactive oxygen species in the microenvironment. The released CsA and SS-31 synergistically restore the mitochondrial membrane potential and the balance between mitochondrial fission and fusion, subsequently protecting neurons from apoptosis and reducing the activation of microglia in the brains of 5 × FAD mice (Figure 4A).

Inorganic and hybrid carriers represent a related branch of delivery-first systems. Metal–organic frameworks (MOFs), Prussian blue-based nanocomplexes, selenium-containing nanoparticles, and other hybrid architectures provide high loading capacity, programmable release, and, in some cases, intrinsic catalytic activity (Ahmadi et al., 2022). Their appeal lies in the possibility of integrating carrier function with therapeutic function. Zhang et al. designed dissolving microneedles combined with nanocarriers are used to enhance nasal-brain drug delivery (Figure 4B). To facilitate nasal administration, toothbrush-shaped microneedle patches were fabricated using hyaluronic acid-coated microneedles and tannic acid-crosslinked gelatin as the base. These patches completely dissolve in the nasal mucosa within a few seconds, leaving only the base, thereby releasing the loaded cyclodextrin-based metal-organic framework (CD-MOFs) without affecting the nasal cilia and nasal microbial communities (Liu Y. et al., 2024). As a high-load cinobufagin nanocarrier, these potassium-structured CD-MOFs, with ergosterol enhancement and lactoferrin functionalization, have good physical stability and good biocompatibility, and can effectively achieve brain-targeted drug delivery, significantly reducing the damage to nerve cells caused by hydrogen peroxide and scopolamine (Zhang et al., 2023). Still, compared with organic systems, inorganic or hybrid nanosystems face sharper questions regarding long-term persistence, metal ion leakage, degradation products, and manufacturability under clinical-quality conditions (Huang F. et al., 2024; Li et al., 2025; Meng et al., 2024).

### 5.2 Catalytic antioxidant nanozymes and redox-regulating nanomaterials

A second major category comprises catalytic nanosystems in which the nanoparticle itself is not merely a vehicle but an active therapeutic entity (Li Z. et al., 2022). This approach is particularly relevant to AD because mitochondrial oxidative stress is both an early event and a self-amplifying driver of amyloid toxicity, tau pathology, synaptic failure, and neuroinflammation (Wu et al., 2025). Unlike conventional antioxidants, which are rapidly consumed and often show poor brain bioavailability, nanozymes can provide sustained enzyme-like catalytic activity and may continuously decompose superoxide, hydrogen peroxide, or lipid peroxides in the diseased microenvironment (Xu et al., 2024).

CeO_2_ nanoparticles, due to their reversible Ce^3+^/Ce^4+^ redox cycle, can mimic SOD and CAT activity, effectively scavenging ROS, thereby protecting mitochondria from oxidative damage (Wei et al., 2025). The CeO_2_/curcumin composite nanosystem developed by Han et al. releases CeO_2_ nanoparticles at AD lesion sites through a ROS-responsive release mechanism (Yang P. et al., 2020). These nanoparticles can be phagocytosed by activated microglia, and after entering cells, exert sustained antioxidant effects, protecting mitochondria from ROS damage, thus improving mitochondrial function. However, this study did not directly target mitochondria, and reliance on ROS to trigger release may limit efficacy. Whereas in the study by H. J. Kwon et al. TPP-modified CeO_2_ nanoparticles were used to directly target mitochondria, utilizing the mitochondrial membrane potential to achieve precise targeting (Kwon et al., 2016). Experiments confirmed that TPP-ceria nanoparticles could effectively accumulate in mitochondria, scavenge mitochondrial ROS, repair damaged mitochondrial cristae structures, and significantly improve mitochondrial membrane potential, providing a direct mitochondrial protection strategy for AD treatment. Additionally, Patricia Gutiérrez-Carcedo et al. found that gold nanoparticle modification introduced significant changes to the mitochondrial effects of CeO_2_ and upregulated the expression of mitochondrial function-related genes (NRF1 and NFE2L1), thereby enhancing mitochondrial respiration and antioxidant defense (Gutiérrez-Carcedo et al., 2020). The TPP-functionalized AuCeO_2_ (TPP-AuCeO_2_) prepared in this study accumulated near mitochondria, and through efficient ROS scavenging and regulation of mitochondrial function, provided new ideas for AD treatment (Figure 4C).

Beyond ceria, a growing set of catalytic nanomaterials has been explored in AD-related settings, including TPP-MoS2 quantum dots, Cu2-xSe-TPP nanomaterials, palladium-based systems, iron–polyphenol coordination particles, ruthenium oxide nanoparticles, and diselenium nanospheres. The Ren research team and Wang team respectively designed TPP-MoS_2_ nanozymes (TPP-MoS_2_ QDs) and TPP-modified Cu_2-_Se nanozymes (Cu_2-_Se-TPP nanoparticles) (Ren et al., 2020). Both materials possess dual SOD and CAT enzyme activities, effectively scavenging mitochondrial ROS, improving oxidative stress, and significantly alleviating cognitive impairment in AD mice.

Whereas ultrasmall iron-gallic acid coordination polymer nanoparticles (Fe-GA CPNs) clear ROS by mimicking SOD and POD enzyme activities, reduce mitochondrial oxidative stress, and improve neuroinflammation and cognitive function in AD models (Wang L. et al., 2025). Furthermore, ultrasmall RuO_2_ nanoparticles (Ren et al., 2020) with their multi-enzyme activities (SOD, CAT, POD) can effectively scavenge ROS, activate mitophagy, repair damaged neurons, and regulate microglial polarization (Figure 4D).

More recent work on Cu_2_-xSe-TPP similarly indicates that catalytic redox buffering can be coupled to mitochondrial targeting and microglial reprogramming (Zhang et al., 2019). In parallel, selenium-based nanosystems have attracted attention because they may combine glutathione peroxidase-like activity with anti-ferroptotic effects, which is conceptually important in AD where oxidative injury, mitochondrial failure, and iron dysregulation often converge (Jia et al., 2021; Chen Y. et al., 2024). Wang et al. designed and synthesized multifunctional double-selenium 662 nanospheres (CLNDSe) (Wang J. et al., 2023). CLNDSe nanoparticles modified with the A_2A_AR agonist (CGS) exhibited A_2A_AR-targeted delivery across the BBB both *in vitro* and *in vivo* (Huang et al., 2025). After modification with the LPFFD short peptide, CLNDSe nanoparticles effectively inhibited Aβ_42_ aggregation and attenuated Aβ_42_-induced neurotoxicity by inhibiting oxidative damage and mitochondrial dysfunction. The nerve growth factor (NGF) related to the large SE spheres significantly reduced tau phosphorylation and neuronal activation in APP/PS1 mice (Chen Y. et al., 2024). The administration of CLNDSe nanoparticles *in vivo* also effectively restored the antioxidant capacity of GPX1/4, alleviated neuronal loss and neurofibrillary tangles, prevented neurodegeneration, and ultimately improved the cognitive deficits of APP/PS1 mice. Importantly, CLNDSe nanoparticles showed good safety and biocompatibility.

This category has several strengths. First, catalytic nanoparticles may reduce the need for repeated high-dose drug administration because the nanomaterial performs the therapeutic chemistry directly. Second, the same platform can sometimes regulate more than one redox pathway, for example, by lowering mtROS, limiting lipid peroxidation, and indirectly dampening NLRP3 activation. Third, catalytic systems can be paired with imaging or stimulus-responsive functions. However, enthusiasm should be tempered by several caveats. Enzyme-mimetic activity measured in solution does not always translate to the crowded and protein-rich intracellular environment. In addition, nanozyme activity may vary with pH, corona formation, lysosomal sequestration, or oxidation state changes *in vivo*. Finally, the redox biology of AD is context-dependent; complete ROS suppression is not necessarily desirable, because physiological ROS also participate in signaling. Future work should therefore focus less on “maximal scavenging” and more on restoring redox homeostasis within a therapeutic window.

### 5.3 Biomimetic vesicles and membrane-camouflaged systems

#### 5.3.1 Exosomes and their analogues

Exosomes have shown an important role in mitochondrial targeted therapy for AD (Cui et al., 2021; Jung et al., 2025; Xiao et al., 2017). Studies have shown that mitochondrial dysfunction is closely related to oxidative stress and Aβ accumulation in AD patients (Ganguly et al., 2017; Adnan et al., 2025). Exosomes can also be used as carriers to deliver protective molecules (such as mitochondrial function regulatory proteins or specific mirnas) to damaged neurons to improve mitochondrial function (Currim et al., 2024; Polanco et al., 2018). For example, M_2_ microglia-derived exosomes can improve mitochondrial mass and reduce neuronal damage by activating PINK1/Parkin-mediated mitophagy (Li N. et al., 2022). In addition, exosomes can also remove Aβ and reduce mitochondrial oxidative stress, thereby protecting neuronal function (Franco et al., 2022). For example, MSCs-derived exosomes improve mitochondrial function and alleviate cognitive decline by up-regulating brain-derived neurotrophic factor (BDNF) -related pathways (Turac et al., 2018). These findings suggest that exosomes provide novel targets for AD treatment by regulating mitophagy and oxidative stress.

As natural carriers, engineered exosomes have potential applications in AD therapy. Lydia Alvarez-Erviti’s group used targeted exosomes (RVG-exosomes) to deliver siRNAs to mouse brain neurons to achieve specific gene silencing and reduce Aβ toxicity (Alvarez-Erviti et al., 2011). In addition, exosome treatment reduced NLRP3 inflammasome activation and the release of pro-inflammatory factors (such as IL-1β and TNF-α), which indirectly alleviated mitochondrial stress and indirectly improved mitochondrial function. Xu et al. developed mesenchymal stem cell-derived exosomes with high SHP2 expression (MSC-EVs-SHP2) for the treatment of AD (Xu et al., 2022). Exosomes deliver SHP2 to the brain through the BBB, significantly enhance neuronal mitophagy, remove damaged mitochondria, inhibit NLRP3 inflammasome activation, reduce neuronal apoptosis and neuroinflammation, and finally improve cognitive function in AD mice. Studies have highlighted the potential of engineered exosomes in repairing mitochondrial dysfunction, providing a new strategy for AD treatment (Figure 4E).

#### 5.3.2 Mitochondrial membrane biomimetic nanoformulations

Mitochondrial membrane biomimetic nanoparticles achieve precise intervention in the pathological cascade of AD through a dual biomimetic strategy involving the fusion of neuronal membranes with mitochondrial membrane proteins (Huang Y. et al., 2024). The core design features a dual-membrane synergistic targeting system. The carrier surface is coated with neuronal plasma membranes, which incorporate adhesion proteins such as NCAM and SynCAM1, leveraging homologous recognition to enhance BBB penetration efficiency. The inner core contains chimeric mitochondrial inner membrane channel proteins, such as the TOM20/TIM23 complex, which utilize the mitochondrial targeting signal peptide (MTS) to guide the carrier across the double mitochondrial membrane, achieving efficient drug enrichment in the mitochondrial matrix.

Multifaceted synergistic intervention against pathology is accomplished through several functional components loaded onto the carrier. For antioxidation and anti-ferroptosis, the quercetin metal chelating network sequesters free iron ions to inhibit the Fenton reaction while concurrently activating the Nrf2 pathway to upregulate glutathione synthesis, reducing iron deposition and lipid peroxidation in the brains of APP/PS1 mice by 60% and 45%, respectively. Calcium homeostasis is reestablished via the co-delivery of Mg^2+^, which competitively inhibits Ca^2+^ influx, and CypD siRNA, which silences CypD expression; this dual-pathway approach inhibits the abnormal opening of the mPTP, restoring 70% of the membrane potential and reducing the neuronal apoptosis rate by 65%. Furthermore, autophagy is activated by SHP2-overexpressing exosomes that phosphorylate PINK1, enhancing Parkin-mediated ubiquitination and promoting the clearance of damaged mitochondria, thereby increasing Aβ clearance efficiency (Aschrafi et al., 2016). The intelligent delivery and sustained-release profile of the system are enhanced by several innovative technologies. A “nanobrake” design responds to the lesion microenvironment characterized by high MMP-9 expression, activating cell-penetrating peptides for targeted neuronal accumulation, which increases the lesion enrichment rat. The sustained-release system is optimized using a Zn-MOF functionalized alumina membrane (NPAlMs) modified with a PVP/graphene coating, enabling the sustained release of memantine over 28 days; density functional theory calculations verify the enhanced drug-carrier binding energy, resulting in a 40% reduction in systemic toxicity. Additionally, BBB penetration is significantly improved by Ang-2-modified Prussian blue nanoparticles, which increase the drug concentration in the brain by 2.8-fold through receptor-mediated endocytosis via LRP-1 (Blesa et al., 2007).

The current technical bottlenecks mainly lie in the freeze-thaw inactivation of membrane proteins (with an activity loss of over 30%), batch consistency control (with a membrane fusion degree difference of up to 15%), and immunogenicity risks. In the future, artificial intelligence will be used to optimize the folding conformation of membrane proteins (such as AlphaFold predicting the stability of MTS), and dynamic monitoring probes (such as the near-infrared II region diagnostic system developed by Wang’s team, Ag_2_S@PPAR-siSOX9) will be integrated. With the deep intersection of membrane protein engineering and computational materials science, such carriers are expected to drive the treatment of AD from “symptom relief” to a new era of “pathological correction”, providing a milestone solution for reversing the neurodegenerative process (Thangamani et al., 2017).

### 5.4 Nucleic-acid platforms and mitochondrial quality-control reprogramming

**FIGURE 5:**
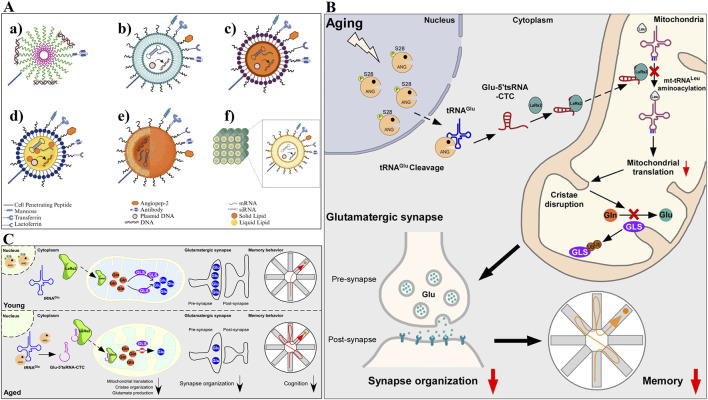
Nucleic-acid nanocarriers and mitochondrial RNA-related regulatory mechanisms relevant to AD and brain aging. **(A)** Structural depiction of different types of nanoparticles along with functionalization strategies for nucleic acid delivery: (a) Polymeric micelles; (b) Liposomes, (c) Solid lipid nanoparticles (SLNs), (d) Nanostructured lipid carriers, (e) Polymeric nanoparticles, and (f) Cubosomes (Chinenye et al., 2024). Copyright 2024, Frontiers. **(B)** Aging induces dephosphorylation of angiogenin, leading to its mislocalization in the cytoplasm to cleave tRNAGlu. Mitochondrial accumulation of the cleavage product Glu-50 tsRNA-CTC impairs mitochondrial translation, cristae organization, and glutaminase-dependent glutamate biogenesis. Thus, Glu-50 tsRNA-CTC accelerates brain aging and ASO targeting Glu-50 tsRNA-CTC rescues age-related phenotypes in mice (Li et al., 2024). Copyright 2024, Elsevier. **(C)** The working model of ASO targeting Glu-50 tsRNA-CTC to rescue agerelated phenotypes in mice (Li et al., 2024). Copyright 2024, Elsevier.

Nucleic-acid nanomedicine provides a strategy to regulate upstream molecular pathways that drive mitochondrial dysfunction in AD (Hoque et al., 2023; Valiukas et al., 2022). In this approach, nanocarriers deliver siRNA, antisense oligonucleotides (ASOs), miRNA mimics/inhibitors, or other gene-regulating cargos to modulate pathological processes such as Aβ production, mPTP opening, neuroinflammation, defective mitophagy, and impaired mitochondrial translation (Chen T. et al., 2024). Various delivery platforms, including polymeric micelles, liposomes, solid lipid nanoparticles, nanostructured lipid carriers, polymeric nanoparticles, and cubosomes, have been developed to improve nucleic-acid stability, cellular uptake, brain delivery, and surface functionalization (Chinenye et al., 2024) (Figure 5A).

Among these approaches, siRNA-based systems are the most widely explored. For example, BACE1 siRNA-loaded nanocomplexes can reduce amyloidogenic Aβ production, while CypD-, NF-κB-, or ROCK2-targeted nucleic-acid platforms may respectively inhibit mPTP opening, suppress inflammatory mitochondrial injury, and improve autophagosome trafficking or mitophagy-related quality control (Belenichev et al., 2025). Compared with conventional antioxidant delivery, these strategies attempt to reprogram the mitochondrial damage network at the gene-regulatory level and may therefore be more suitable for the multifactorial pathology of AD (Singh et al., 2024).

Recent evidence also links nucleic-acid regulation to mitochondrial dysfunction during brain aging, a major risk context for AD (Zhang et al., 2023). Aging-associated angiogenin mislocalization can induce cleavage of tRNA^Glu and generate Glu-50 tsRNA-CTC, which accumulates in mitochondria and impairs mitochondrial translation, cristae organization, glutamate metabolism, synaptic organization, and memory function (Figure 5B). ASO-mediated inhibition of Glu-50 tsRNA-CTC was shown to rescue age-related mitochondrial and cognitive phenotypes in mice (Figure 5C), suggesting that mitochondrial RNA-related pathways may represent emerging intervention targets for AD-associated mitochondrial vulnerability (Li et al., 2024).

Despite their promise, nucleic-acid nanoplatforms remain at an early translational stage. Major challenges include nuclease-mediated cargo degradation, endosomal trapping, off-target gene silencing, innate immune activation, inefficient delivery to the aging human brain, and insufficient validation of durable mitochondrial rescue (Tang et al., 2024). Future studies should demonstrate not only target knockdown, but also functional recovery of mitochondrial respiration, membrane potential, mitophagy flux, synaptic integrity, and cognitive performance.

### 5.5 Alternative administration routes and key translational barriers

Most mitochondria-targeted AD nanomedicines have been developed for intravenous administration and trans-BBB transport (Wu et al., 2023). This remains the dominant paradigm, but it should not be presented as the only relevant route (Wang et al., 2020; Nakhla et al., 2025). Intranasal delivery has become an important complementary strategy because it can partially bypass the BBB through olfactory and trigeminal pathways, reduce first-pass metabolism, and improve patient acceptability for chronic therapy. Recent reviews emphasize that intranasal nanoformulations can enhance nasal residence time, improve mucosal penetration, and provide relatively direct brain access, although they still face challenges including unstable absorption, mucociliary clearance, local irritation, and formulation complexity (Chinenye et al., 2024). Intrathecal or intracerebroventricular administration is more invasive, but may remain relevant for selected biologics or nucleic-acid payloads when systemic delivery is inadequate. Therefore, future reviews of AD mitochondrial nanotherapy should explicitly acknowledge route selection as part of strategy design rather than treating it as a secondary formulation issue.

More broadly, the translational bottlenecks of the field are now clearer than the efficacy literature sometimes suggests. First, safety remains undercharacterized. For many systems, especially inorganic or hybrid nanoparticles, long-term biodistribution, degradation, and clearance in the brain, liver, spleen, and kidney are insufficiently mapped (Li et al., 2023). Second, immunological behavior is often simplified; protein corona formation, complement activation, and microglial responses may substantially alter performance after repeated dosing. Third, manufacturing complexity is a serious barrier (Guo et al., 2024). Multi-ligand, multi-component nanoparticles may perform well in small laboratory batches but prove difficult to reproduce under Good Manufacturing Practice conditions. Recent translational reviews in CNS nanomedicine increasingly stress the need for quality-by-design chemistry, manufacturing and controls, robust PK/PD modeling, and long-term safety monitoring from the outset rather than as late-stage add-ons (Liu et al., 2025).

A further issue is validation of true mitochondrial targeting. Many reports infer mitochondrial delivery from colocalization images alone, yet these do not always distinguish endolysosomal proximity from genuine intramitochondrial accumulation (McGill Percy et al., 2025). For a field centered on subcellular precision, stronger validation standards are needed, including orthogonal imaging, biochemical fractionation, and functional mitochondrial readouts. Finally, the disease models themselves require caution. Rapid-onset transgenic mouse models capture selected aspects of amyloid pathology but do not fully reproduce the aging-associated, vascular, metabolic, and inflammatory complexity of sporadic AD. A nanomedicine that works in a short-term amyloid model has not necessarily solved the translational problem of human AD (Pahal et al., 2025; Asimakidou et al., 2024).

Taken together, the current landscape suggests that the next advance in mitochondrial nanotherapy for AD will likely come not from simply creating more complex nanoparticles, but from converging on simpler, mechanism-driven, and manufacturable systems with validated subcellular delivery. In practical terms, the most promising future platforms are likely to be those that combine a clinically realistic administration route, a minimally sufficient targeting architecture, a disease-relevant mitochondrial mechanism, and a transparent translational plan.

## 6 Conclusion

This review has provided a synthesized reappraisal of mitochondrial dysfunction as an early and sustained driver of AD pathology, and has consolidated recent advances in nanomedicine as a uniquely equipped strategy to address this therapeutic gap. The evidence reviewed herein demonstrates that impairments in mitochondrial bioenergetics, quality control, and dynamics are not merely downstream consequences of amyloid-beta and tau pathology but rather represent a central nexus that initiates and amplifies the neurodegenerative cascade. Conventional pharmacological approaches have largely failed to address this foundational dysfunction owing to the dual barriers of the BBB and the mitochondrial double membrane. In contrast, the diverse nanotherapeutic platforms evaluated in this review-including organic and inorganic nanocarriers, nanozymes, biomimetic vesicles, and gene-modulating systems-exhibit the capacity to sequentially overcome these barriers and achieve subcellular precision targeting of neuronal mitochondria, thereby restoring redox balance, enhancing mitophagy, and preserving adenosine triphosphate synthesis at the site of injury.

While preclinical studies have yielded encouraging results, the clinical translation of mitochondria-targeted nanomedicine remains contingent upon addressing several critical challenges. We propose that future research prioritize three key directions. First, the translational bottlenecks of scalable manufacturing, long-term biocompatibility assessment, and comprehensive *in vivo* metabolic profiling must be systematically resolved to enable regulatory approval. Second, the development of multifunctional nanoplatforms that integrate mitochondrial restoration with parallel disease-modifying strategies-such as amyloid-beta clearance, tau modulation, and anti-neuroinflammation-offers a rational path toward synergistically tackling the multifactorial pathogenesis of AD. Third, as disease subtyping and biomarker-guided stratification advance, the field must pivot toward personalized nanotherapeutic regimens tailored to specific stages and molecular subtypes of AD, accompanied by rigorous evaluation in clinically relevant large-animal models and ultimately human trials. By addressing these priorities, mitochondria-targeted nanomedicine holds the potential to evolve from a promising preclinical concept into a requisite disease-modifying paradigm capable of intercepting the earliest triggers of AD.
